# Distribution, Persistence and Interchange of Epstein-Barr Virus Strains among PBMC, Plasma and Saliva of Primary Infection Subjects

**DOI:** 10.1371/journal.pone.0120710

**Published:** 2015-03-25

**Authors:** Hin Kwok, Koon Wing Chan, Kwok Hung Chan, Alan Kwok Shing Chiang

**Affiliations:** 1 Department of Paediatrics & Adolescent Medicine, Li Ka Shing Faculty of Medicine, The University of Hong Kong, Queen Mary Hospital, Pokfulam, Hong Kong; 2 Department of Microbiology, Li Ka Shing Faculty of Medicine, The University of Hong Kong, Queen Mary Hospital, Pokfulam, Hong Kong; University of Sussex, UNITED KINGDOM

## Abstract

Our study aimed at investigating the distribution, persistence and interchange of viral strains among peripheral blood mononuclear cells (PBMC), plasma and saliva of primary Epstein-Barr virus (EBV) infection subjects. Twelve infectious mononucleosis (IM) patients and eight asymptomatic individuals (AS) with primary EBV infection were followed longitudinally at several time points for one year from the time of diagnosis, when blood and saliva samples were collected and separated into PBMC, plasma and saliva, representing circulating B cell, plasma and epithelial cell compartments, respectively. To survey the viral strains, genotyping assays for the natural polymorphisms in two latent EBV genes, EBNA2 and LMP1, were performed and consisted of real-time PCR on EBNA2 to distinguish type 1 and 2 viruses, fluorescent-based 30-bp typing assay on LMP1 to distinguish deletion and wild type LMP1, and fluorescent-based heteroduplex tracking assays on both EBNA2 and LMP1 to distinguish defined polymorphic variants. No discernible differences were observed between IM patients and AS. Multiple viral strains were acquired early at the start of infection. Stable persistence of dominant EBV strains in the same tissue compartment was observed throughout the longitudinal samples. LMP1-defined strains, China 1, China 2 and Mediterranean+, were the most common strains observed. EBNA2-defined groups 1 and 3e predominated the PBMC and saliva compartments. Concordance of EBNA2 and LMP1 strains between PBMC and saliva suggested ready interchange of viruses between circulating B cell and epithelial cell pools, whilst discordance of viral strains observed between plasma and PBMC/saliva indicated presence of viral pools in other undetermined tissue compartments. Taken together, the results indicated that the distribution, persistence and interchange of viral strains among the tissue compartments are more complex than those proposed by the current model of EBV life cycle.

## Introduction

Epstein-Barr virus (EBV) is a ubiquitous human herpesvirus infecting more than 90% of the adult human populations. Most populations in the world are infected by EBV within the first few years of life. The majority of childhood infections are asymptomatic. Only a minority of the infected individuals will develop a clinical syndrome known as infectious mononucleosis (IM), which is characterized by fever, enlarged neck lymph nodes, tonsillopharyngitis and fatigue.

Two major types of EBV, namely type 1 and type 2 (also known as types A and B), are distinguished by sequence polymorphisms in Epstein-Barr nuclear antigen (EBNA)-2, -3A, -3B and -3C genes [1,2]. They occur worldwide but with different geographical distribution. EBV type 1 (EBV1) predominates in European, American, Chinese and South-east Asian populations whilst EBV type 2 (EBV2) can only be detected in a minority of healthy carriers in these populations [3]. On the other hand, a more even distribution of EBV1 and -2 was observed in African and Papua New Guinean populations [4,5]. Classification by the two EBV types, however, is not sufficient in revealing the extensive strain diversity of EBV in clinical samples, and thus, only allows limited understanding of EBV epidemiology. Sequence of EBNA2 gene in EBV1 was found to harbor stable and constant nucleotide changes [4]. Further analysis of EBNA2 and Epstein-Barr encoded RNA (EBER) gene sequences of EBV genomes contained in clinical samples of different malignant and benign EBV-associated diseases identified common variant strains of EBV1, namely, groups 1, 2, 3a, 3b and 3e [6]. Another highly polymorphic EBV gene, namely LMP1, is frequently used in molecular epidemiological studies of EBV infection. The loss of the XhoI restriction site from exon 1 of LMP1 gene was first reported in the CAO cell line derived from a case of Chinese nasopharyngeal carcinoma (NPC) [7]. A 30-bp deletion, at the 3’ terminal region of LMP1, which causes the loss of 10 amino acids, was detected in EBV genomes contained in NPC specimens from Taiwan and South China [7,8]. Different patterns of LMP1 sequence variations were later recognized in NPC samples and EBV-infected lymphoid cell lines and were designated China 1, China 2, and Alaskan strains [9,10]. Two more strains, Mediterranean and North Carolina, were also found when a larger panel of cell lines and clinical samples of EBV-associated malignancies and non-malignant conditions such as IM were analyzed [11]. Mediterranean strain is further classified into M+ and M-, indicating the presence (+) or absence (-) of the LMP1 30-bp deletion. Hence, a total of seven LMP1 strains, including the prototype B95–8 strain, were defined.

Earlier studies of EBV variants contained in blood and throat washings of healthy long term viral carriers used spontaneous lymphoblastoid cell lines (LCL) transformed by viruses rescued from these clinical samples and concluded that each individual only harbors one dominant strain of virus [12]. Dual infections were later detected in clinical specimens of healthy individuals [13] and of NPC patients [14], using restriction fragment length polymorphism (RFLP) analysis of *BamHI* F fragment of EBV DNA. Multiple EBV variants were subsequently detected in immunocompromised individuals, such as in human immunodeficiency virus (HIV)-positive subjects [15,16] and in immunocompetent healthy donors [17–19], using PCR amplification of different polymorphic genes of EBV genomes contained in clinical specimens. In other studies, multiple EBNA1 subtypes could also be detected in peripheral blood mononuclear cells (PBMC) and throat washings of healthy donors [20,21] and in those of NPC patients [22].

Heteroduplex tracking assay (HTA) is a sensitive technique which can differentiate sequences with small number of differences and is capable of revealing multiple co-existing strains in viral infections. It has been used to analyze the evolution of human immunodeficiency virus (HIV) strains in patients who develop resistance to viral therapy [23]. In EBV genotyping studies, HTA served to distinguish the five EBNA2 groups and the seven LMP1 strains [24,25]. With use of HTA, co-infection of multiple EBV strains was found in asymptomatic carriers and infectious mononucleosis [18,24,26]. Nonradioactive HTA has been developed for detection of variants in *Plasmodium falciparum*, using digoxigenin (DIG) label as the detection system. [27]. In our study of EBV strains, 6-FAM fluorescent-label and direct fluorescent detection by ABI PRISM 377 automated sequencer were used to further reduce the steps for signal detection.

The current understanding of EBV biology states that the virus infects both B cells and epithelial cells. Whether multiple EBV strains can persist in or freely interchange between PBMC (circulating B cell compartment), plasma and saliva (epithelial cell compartment) over time is still unclear. In this longitudinal study, EBV strains harbored in cryopreserved serial samples of PBMC, saliva and plasma from a cohort of children with serologically confirmed primary EBV infection were analyzed, using typing assays (EBV1 and 2 and LMP1 deletion) and HTA (EBNA2 and LMP1). The following questions were asked: Are multiple EBV strains acquired at the start of primary infection? Do these strains persist in individual tissue compartments and do they change within these compartments over time? Are the strains similar or different between the tissue compartments? Any interchange of viral strains between the tissue compartments? These are questions that are pertinent to further understanding of the natural distribution and interchange of multiple EBV strains within individual viral carriers.

## Methods and Materials

### Ethic statement

Clinical specimens were collected at Department of Paediatrics and Adolescent Medicine, The University of Hong Kong, Queen Mary Hospital, between 2003 and 2006, after obtaining informed written consent from the parents. The study is approved by Institutional Review Board of The University of Hong Kong/Hospital Authority Hong Kong West Cluster for the purpose of infectious mononucleosis studies.

### Patients

Two study cohorts consisting of twelve children with infectious mononucleosis (IM) and eight with asymptomatic primary infection (AS) were included. Children of age 18 or below with symptoms and signs such as fever, cervical lymphadenopathy, tonsillopharyngitis and fatigue, were recruited into the study as IM subjects. The AS subjects were identified serendipitously by serological screening of children admitted to the general pediatric wards of Queen Mary Hospital, Hong Kong. The serological profile of primary EBV infection is defined as VCA IgM positive or negative, VCA IgG positive (low avidity) and EBNA negative. Peripheral blood samples and saliva were collected at the time of clinical diagnosis (IM) or serological screening (AS) and at 1 week, 1 month, 3 months, 6 months and 12 months after the diagnosis (for IM) or screening (for AS).

### Clinical samples and processing

Five to ten milliliter of blood was collected from each patient at each time point. After centrifugation, PBMC were isolated by standard Ficoll-Hypaque density gradient method. Cells were resuspended in 10% DMSO, 90% fetal bovine serum for storage. Plasma portion was further centrifuged at 13000 rpm for 10 minutes followed by collection of supernatant to ensure that cell-free plasma was obtained. The patient was also asked to chew for 1 minute on a cotton wool cylinder of a salivette (Sarstedt, Nümbrecht, Germany), from which saliva was extracted by centrifugation at 2000 rpm for 10 minutes. PBMC, plasma and saliva samples were all cryopreserved until use.

### Reference cell lines

Cell lines used as controls in our assays include B95–8 [28], Akata [29], Namalwa [30] and Jijoye [31]. DNA was extracted from these cell lines. B95–8 contains type 1 EBV genome and does not have the 30 bp deletion at the LMP1 locus, hence, was used as LMP1 wild-type non-deleted control for LMP1 typing assay. Akata has the 30-bp deletion in LMP1 gene and was used as LMP1 deletion control. Namalwa contains 2 copies of type 1 EBV genomes per cell and was used as type 1 control for EBNA2 real-time PCR typing assay. Jijoye harbors a type 2 EBV genome and was used as type 2 control for EBNA2 real-time PCR typing assay. All the above cell lines were cultured, under aseptic condition, in RPMI-1640 (Gibco, Invitrogen, NY, USA) supplemented with 10% FBS (Gibco BRL, USA) at 37°C in 5% CO_2_.

### DNA isolation

DNA from PBMC was extracted by Qiagen DNeasy Blood & Tissue kit (QIAGEN, Hilden, Germany). DNA from plasma and saliva was extracted by QIAamp DNA Blood Mini Kit (QIAGEN).

### Quantitative PCR for EBV loads in PBMCs, plasma and saliva

Quantitative PCR assays for EBV DNA in PBMCs were performed on ABI PRISM 7900 sequence detector (Applied Biosystems, Life Technologies, USA). EBV loads in PBMCs were determined by amplifying EBV DNA polymerase (Pol, BALF5) gene and the input cell quantity was determined by amplifying the human beta-2 microglobulin gene, as described previously [32]. Namalwa cells were used as the standard. EBV loads in plasma and saliva were determined by amplifying the EBV BamH1W repeats of the EBV genome to achieve higher sensitivity [33].

### Overall scheme of genotyping assays

This study utilized two typing assays, EBNA2 typing assay to discern type 1 and type 2 EBV and LMP1 typing assay to discern LMP1 with or without the 30-bp deletion at the 3’ region. We also employed two heteroduplex tracking assays (HTA) to distinguish the different type 1 EBNA2 and LMP1 variants. The EBNA2 variants were classified as group 1, 2, 3a, 3b and 3e. [6] and the LMP1 variants as China 1 (C1) and Mediterranean + (M+) (both have the 30-bp deletion) and China 2 (C2), Mediterranean—(M-), Alaskan (AL), North Carolina (NC) and B95–8 (all without 30-bp deletion) [11]. The details of each typing assay and HTA are described below.

### EBNA2 real-time PCR typing

Real-time PCR typing assay was performed by ABI PRISM 7700 Sequence Detector (AppliedBiosystem, USA). A common primer E2C (EBV coordinates 36522–36541, AGGGATGCCTGGACACAAGA) was used as forward primers for both EBV types. The type-specific reverse primers for type 1 and 2 EBV were E2QR1 (36607–36628, TTAGCCATCCAAAGCATTCGCA) and E2QR2 (36549–36526, TTCTAAGAAGGTATTGAGCCATGC), respectively. The 6-FAM-labeled E2_T1 (36751–36770, 6-FAM-TTGTGACAGAGGTGACAAAA) and the VIC-labeled E2_T2 (36728–36709, VIC-TTGAAGAGTATGTCCTAAGG) were used as probes to type 1 and 2 EBV genome, respectively. The reaction mixture were as follows: 12.5 μl TaqMan Universal PCR Master Mix (Applied Biosystems, Branchburg, New Jersey, USA), 0.25 μl E2C primer (10 μM), 0.25 μl E2QR1 primer (10 μM), 0.25 μl E2QR2 primer (10 μM), 0.25 μl E2T1 probe (10 μM), 0.25 μl E2T2 probe (10 μM), 6.5 μl of distilled water and 5 μl of DNA with concentration of 50 ng/μl.

### LMP1 30-bp deletion typing

PCR was performed to amplify the loci of 30bp deletion in the 3’region of LMP1. The forward and reverse primers were designated LMPdelF (EBV coordinates 168369–168389, AGCGACTCTGCTGGAAATGAT) and FUE_FAM (EBV coordinates 168163–168183, 6-FAM-GTCATAGTAGCTTAGCTGAAC), respectively. GeneScan function of ABI PRISM 377 Automatic Sequencer (AppliedBiosystem, Foster City, CA, USA) was used to separate FAM-labeled PCR products according to their size differences. 30-bp LMP1 variants migrate faster than the undeleted wild type variants, resulting in separation of the two variants. Gel mixture (7%) for 36cm plate was prepared as follows: 6ml of 5X TBE buffer, 4.2 ml of 10X Long Ranger solution, 19.8ml of MilliQ water, 15 μl of TEMED and 150 μl of 10% ammonium persulphate (APS). 2.5 μl mixtures of loading buffer and sample DNA annealed with FAM-labeled probe were loaded. The GeneScan was run with 1x TBE as the running buffer using the ABI PRISM 377 Sequencer (Applied Biosystem, USA) at 48°C.

### Heteroduplex tracking assays (HTA)

Probe for EBNA2 HTA was synthesized by PCR using the group 2 control plasmid (provided by Dr R.J.Tierney, Cancer Research Institute for Cancer Studies, University of Birmingham). The forward and reverse primers are E2SEQ7.1a (EBV coordinates 36671–36690, GCCACAAGGCCCACAAACAG) and E2SEQ8.1_FAM (36901–36920, 6-FAM-AGGCCTTTGTAGTACCGTGA) respectively. Probes for LMP1 HTA were synthesized by PCR using the control plasmids cloned with segments of LMP1 of the C2 and M+ strains (provided by Prof. Nancy Raab-Raub, The University of North Carolina at Chapel Hill, USA). The primers used are LMPdelF (167912–167932, AGCGACTCTGCTGGAAATGAT) and FUE_FAM (167705–167725, 6-FAM-GTCATAGTAGCTTAGCTGAAC). The 6-FAM labeled probe was then gel purified by QIAEX II Gel Extraction Kit (QIAGEN). Condition for the PCR reaction is as follows: 50°C for 2 minutes, 95°C for 10 minutes, repeat 45 times of 95°C for 15 seconds and 60 for 1 minute.

Nested PCR on sample DNA was performed. Primers for the first round of PCR for EBNA2 HTA were E2C and E2SEQ4.1 (37086–37067, GTAATGGCATAGGTGGAATG), and the second round primers were E2SEQ7.1a and E2SEQ8.1a (36901–36920, AGGCCTTTGTAGTACCGTGA).

First round PCR primers for LMP1 HTA are LMP3UT-Eco (167559–167579, atcacgaggaattcAATGTGGCTTTTCAGCCTAGA) and FUE_Eco (167705–167725, atcacgaggaattcGTCATAGTAGCTTAGCTGAAC). The second round primers were LMPdelF and FUE_Eco. The condition was the same for first and second round PCR and used the same condition as probe synthesis. PCR products were gel-purified by QIAEX II Gel Extraction Kit (QIAGEN).

E2SEQ7.1a and E2SEQ8, and LMPdelF and FUE_Eco were used to amplify the reference DNA segment from EBNA2 and LMP1 control plasmids, respectively, by the same PCR condition as above. The annealing reaction mixture was prepared as follows: 1 μl of 6-FAM-labeled probe, 1 μl of annealing buffer (1M NaCl [Amresco, USA], 0.1M Tris [Amresco, USA] pH7.5, 20mM EDTA [Sigma, USA]), 8 μl of nested PCR product. The reaction mixture was heated at 100°C for 5 minutes and then put immediately in ice for 4 minutes. The same conditions were employed to produce heteroduplexes for controls, where gel purified PCR products amplified from control plasmids were used instead of nested PCR product. Both C2 and M+ probes were employed in LMP1 HTA to verify the identity of the LMP1 strains. ABI PRISM 377 Automatic Sequencer was employed in both EBNA2 and LMP1 HTAs. Samples were run in 7% polyacrylamide gel together with the GeneScan Rox 400 ladder (Life Technologies, Grand Island, NY, USA). 36°C was set for pre-run and GeneScan for LMP1 HTA while 48°C was set for EBNA2 HTA.

### Sequencing analysis

Sequencing analysis was performed on about 10% of the PCR products of LMP1 HTA to validate the HTA result. PCR products from these samples were sequenced in both forward and reversed direction with primers LMPdelF and FUE respectively. These PCR products were gel purified by QIAEX II gel extraction kit (QIAGEN) to remove non-specific products and primer dimers. BigDye Terminator v3.1 Cycle sequencing kit (Applied Biosystems, USA) was used and the reaction mix was as follows: 1 μl BigDye Ready reaction mix, 1.5 μl BigDye sequencing buffer, 1μl of 1.6 μM sequencing primer, 6.5 μl of water and PCR product. The reaction was performed by GeneAmp PCR system 9700 (Applied Biosystems, USA) with the following reaction conditions: 96°C for 1 minute, then repeating cycle of 96°C 10 seconds, 52°C 5 seconds and 60°C 4 minutes for 25 times. The samples were sequenced in Genome Research Centre of the University of Hong Kong on 3100 Genetic Analyzer (Applied Biosystems Division; Perkin-Elmer Cetus).

## Results

### Typing and heteroduplex tracking assays of EBNA2 and LMP1 genes

**Fig 1:**
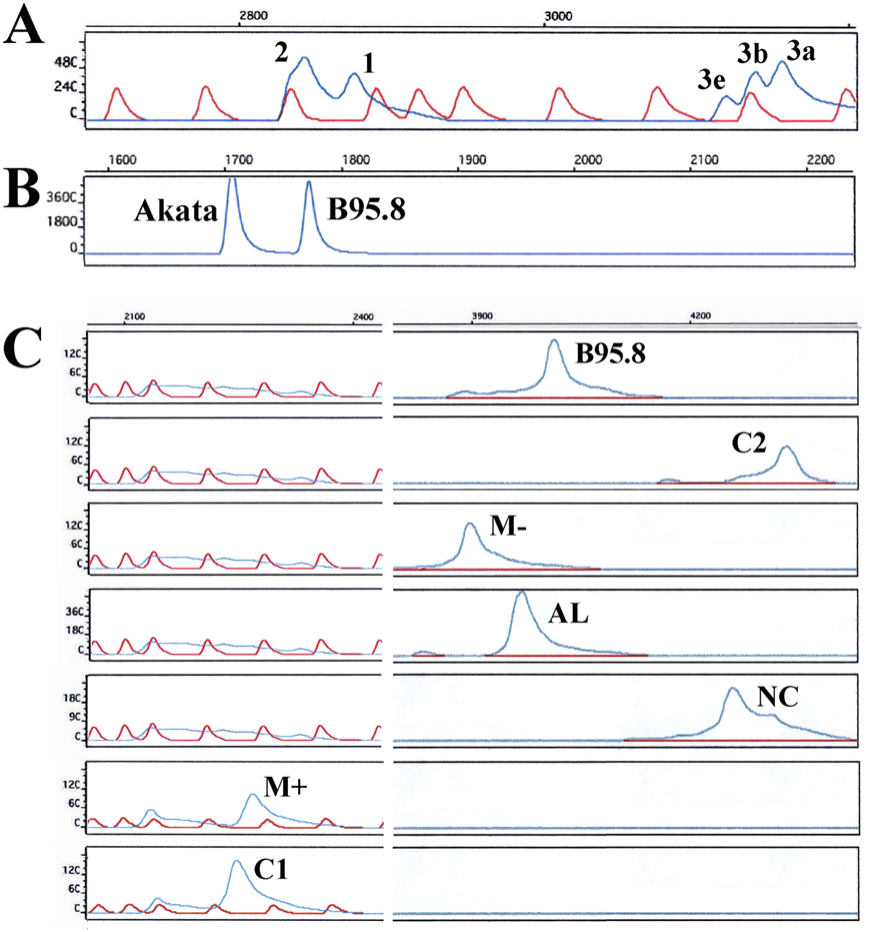
Controls and references for EBNA2 HTA, LMP1 typing and LMP1 HTA. (A) The references of EBNA2 HTA. Each of the five peaks represents a reference strain defined using EBNA2 polymorphisms as defined by Schuster et al [6]. A ROX ladder was loaded to the same lane to indicate the relative positions of the peaks. Since group 2 reference is used as the probe, group 2 strains will be visualized as the peak with highest mobility (left most), followed by group 1 peak. Group 3e, 3b and 3a have lower sequence similarities to those of group 2, and hence, formed heteroduplexes of lowest mobility (right most). (B) Controls for LMP1 typing assay. PCR products from Akata and B95.8 DNA represent the 30-bp deleted and non-deleted LMP1 strains, respectively. Sample DNA will be PCR-amplified by 6-FAM labeled primers and loaded into lanes adjacent to the controls. Positions of the peaks will be directly compared to those of the controls to determine the results of the LMP1 genotyping. (C) The references of LMP1 HTA. Each of the seven peaks represents a reference strain defined using LMP1 polymorphisms as defined by Edwards et al [11]. This figure illustrated the clear separation of the seven reference LMP1 plasmids by the HTA using 6-FAM-labeled PCR product of M+ reference plasmid as the probe.

An LMP1 typing assay was devised to differentiate the 30-bp deleted and non-deleted wild-type. FAM-labeled PCR products were separated by electrophoresis and visualized on ABI PRISM 377 Automatic Sequencer. Real-time PCR-based typing assay was performed to differentiate type 1 and 2 EBV, as defined by EBNA2 gene polymorphisms, using type-specific primers. LMP1-specific HTA [25] and EBNA2 HTA [24] established in previous studies were modified by using a 6-FAM fluorescent label instead of isotopic marker for probe labeling. In LMP1 HTA analysis, samples were analyzed by the use of both C2 and M+ probes for cross-checking. Fig. 1 illustrates the results of LMP1 typing assay and EBNA2 and LMP1 HTA, performed on reference cell lines and plasmids, respectively. Quantitative PCR had been performed on most of the cases and the viral loads expressed in per ml saliva or plasma, or per million PBMCs are shown in S1 Table. Despite the use of nested PCR to increase sensitivity, viral loads of approximately 500 copies per ml DNA were found to be the detection limit of HTA.

### IM and AS cases as cohort of primary EBV infection

EBNA2 and LMP1 typing and HTA were performed on all 20 cases, including 12 IM patients and 8 asymptomatic individuals (AS). There was no observable difference between the strain profiles of symptomatic and asymptomatic individuals.

**Fig 2:**
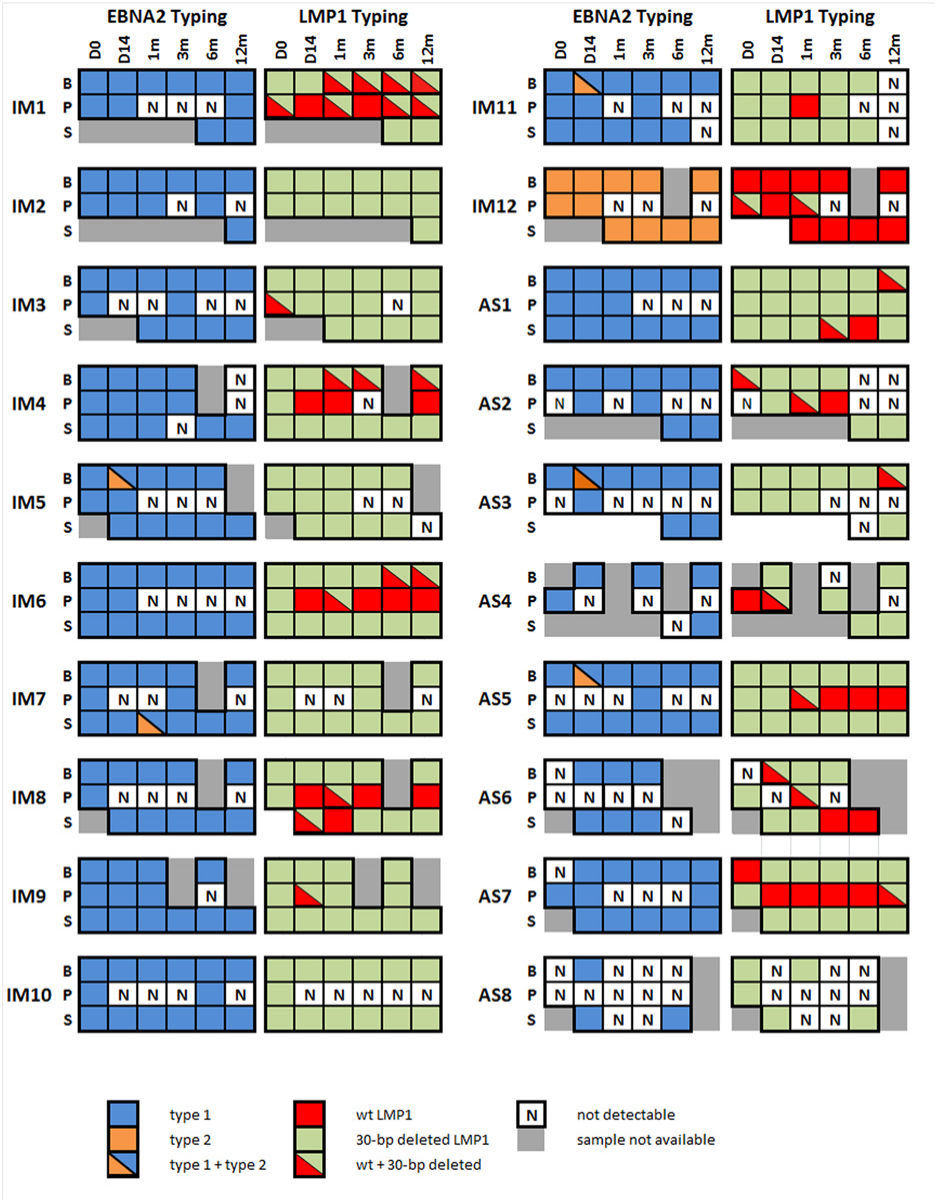
EBNA2 and LMP1 Typing assays on 20 cases. EBV types as defined by EBNA2 (type 1 or 2) and LMP1 (30-bp deleted or wild-type) are illustrated as colored boxes. Each box represents a sample in one of the three compartments, peripheral blood mononuclear cells (B), plasma (P) and saliva (S), at a certain time-point, up to 1 year since diagnosis.

Real-time PCR-based typing assay showed that in 14 out of 20 cases (70%), only type 1 genome was detected (Fig. 2). In four cases (IM5, IM7, IM11 and AS3), type 1 was the predominant strain, yet type 2 was also found co-existing in one to two time-points. In one case, IM12, only type 2 genome was found in all samples. LMP1 30-bp deletion typing assay detected both deleted and non-deleted strains in 75% of all cases (15/20), either co-existed in the same sample, or in a separate manner in samples of different compartment or time points (Fig. 2). Simultaneous detection of both deleted and non-deleted strains tend to be observed in PBMC and plasma instead of in saliva. The saliva compartment of only four cases were detected to have both deleted and non-deleted present and only two out of these four were having both strains co-existing in a single sample at certain time point.

**Fig 3:**
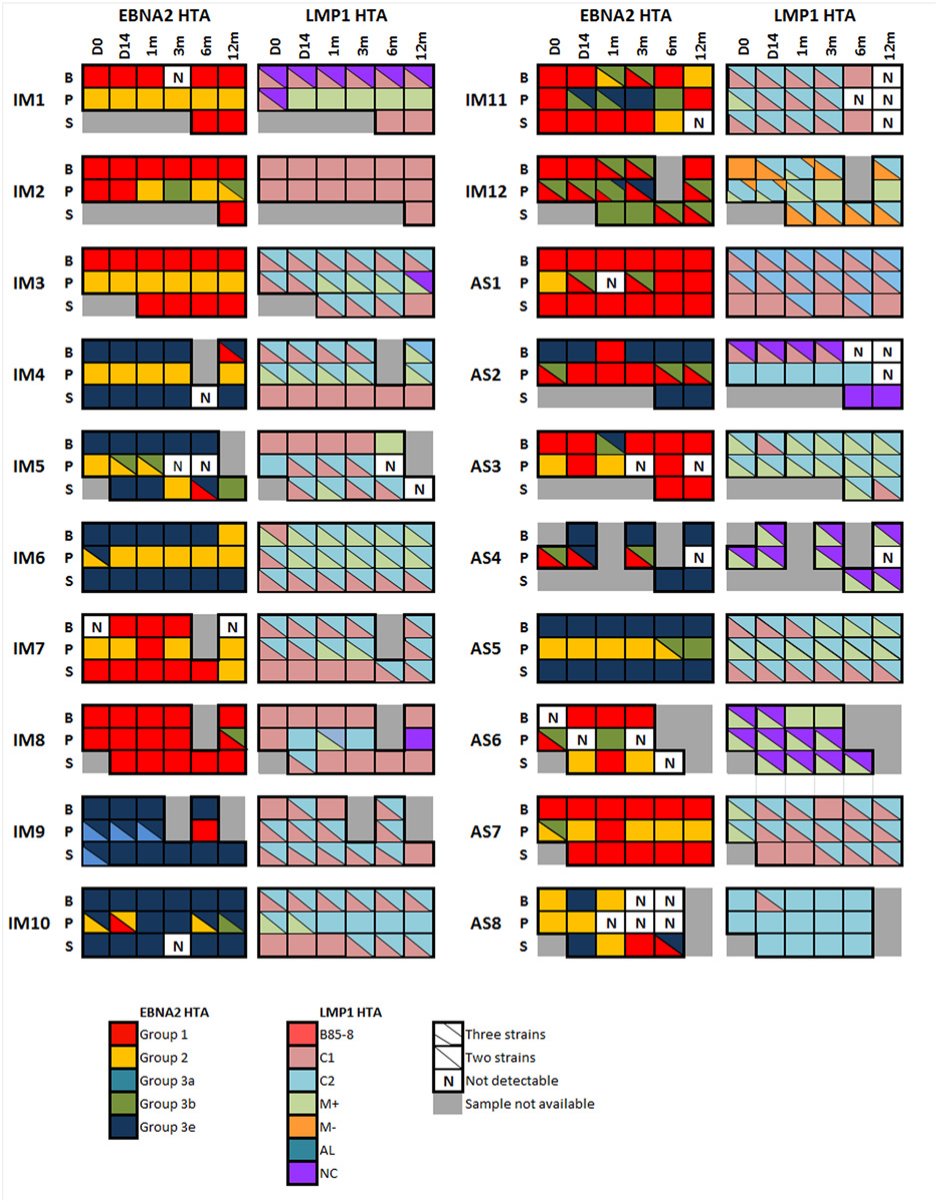
EBNA2 and LMP1 HTA on 20 cases. EBV strains as defined by EBNA2 (group 1, 2, 3a, 3b and 3e) and LMP1 (B95–8, M+, M-, C1, C2, AL, NC) are illustrated as colored boxes. Each box represents a sample in one of the three compartments, peripheral blood mononuclear cells (B), plasma (P) and saliva (S), at a certain time-point, up to 1 year since diagnosis.

All of the five EBNA2 defined groups can be detected with EBNA2 HTA in our cases (Fig. 3). Multiple EBNA2 strains were detected in all 20 cases, with 5 cases having 2 strains, 10 cases having 3 strains and 5 cases with 4 strains (Table 1). Group 1 had been found in 18 out 20 cases (90%), group 2 in 15 cases (75%), group 3b in 13 cases (65%), and group 3e in 12 cases (60%). Group 3a could only be detected in one case (5%). Groups 2 and 3b were found more frequently in plasma than PBMC and saliva samples of the cases (Table 2).

**Table 1:** Number of cases in which a certain number of strains were detected.

EBNA2 typing		EBNA2 HTA		LMP1 typing		LMP1 HTA	
	*no*. *of cases*		*no*. *of cases*		*no*. *of cases*		*no*. *of cases*
type 1 only	14	1 strain	0	wt only	0	1 strain	0
type 2 only	1	2 strains	5	del only	5	2 strains	6
type 1 & 2	5	3 strains	10	wt & del	15	3 strains	12
		4 strains	5			4 strains	2

**Table 2:** Distribution of strains by EBNA2 HTA.

	PBMC	Plasma	Saliva
group 1	13	13	13
group 2	3	13	5
group 3a	0	1	2
group 3b	3	12	2
group 3e	10	12	9

*Number of samples containing the strain.

LMP1 HTA clearly demonstrated the presence of more than one viral strain in all subjects (refer to Fig. 3). Co-infection of four strains was found in two cases (10%), three strains in 12 cases (60%) and two strains in 6 cases (30%) (Table 1). The most common strain found was C1, which was present in 17 cases (85%). C2 in 16 cases (80%), M+ in 15 cases (75%), North Carolina (NC) in six cases (30%), and M- in only one case. None of the cases harbored Alaskan (AL) or B95.8 strain. C1 were found more frequently in PBMC and saliva than plasma samples, while M+ were observed more frequently in plasma (refer to Table 3). The LMP1 HTA results corresponded to those of LMP1 typing assay but showed a greater diversity. This likely reflects a difference of sensitivity between the assays.

**Table 3:** Distribution of strains by LMP1 HTA.

	PBMC	Plasma	Saliva
C1	17	11	15
C2	13	16	15
M+	9	14	4
M-	1	1	1
NC	4	4	3
B95–8	0	0	0
AL	0	0	0

*Number of samples containing the strain.

### Multiple EBV strains acquired in early infection

**Fig 4:**
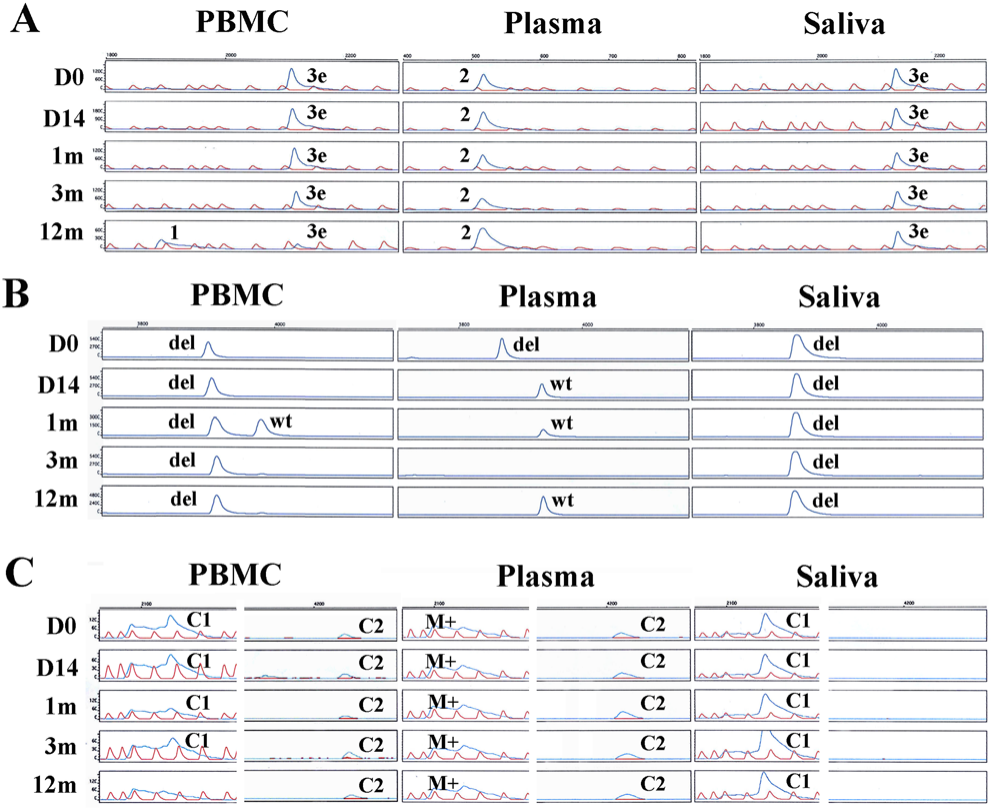
LMP1 typing, EBNA2 HTA, LMP1 typing and HTA of IM4. (A) EBNA2 HTA (B) LMP1 typing in panel D, E and F (C) LMP1 HTA in panel G, H and I. Red peaks represent the ROX ladder. The first rows of EBNA2 HTA and LMP1 typing profiles show the controls. The strains detected were labeled beside the corresponding blue peaks. D0, D14, 1m, 3m, 6m and 12m represents time points of day 0, day 14, 1 month, 3 months, 6 months and 12 months, respectively. A summary of strains detected in this case is in Fig. 2 and 3.

The analyzed profiles of three assays, EBNA2 and LMP1 HTA, and LMP1 typing assay, performed on case IM4, are illustrated in Fig. 4. Group 3e and group 2 were the major groups found in this case by EBNA2 HTA. LMP1 variants C1, M+ and C2 were commonly detected at multiple time-points in PBMC and saliva.

Multiple strains can be captured by looking across different compartment through the combination of different assays. EBNA2 defined group 3e and group 2 are found at day 0, in PBMC and saliva, and in plasma, respectively. At the sample time-point, 30-bp deleted (del) strain was observed in all three compartments, while M+, C1, and C2 were detected by LMP1 HTA. The two HTAs have already shown the presence of multiple strains in a case at the first time-point. Two strains, M+ and C2, were seen in early time-points of plasma samples of IM4 by LMP1 HTA. Similarly, in PBMC, we observed the presence of group 3e and del strain, yet C1 and C2 is found by LMP1 HTA. IM4 was not the only case which multiple EBV strains have been found in early time-points. Multiple strains were also found at day 0 of 17 cases, as illustrated by at least one of the typing assays or HTAs. When both HTAs are considered jointly, the analyzed result may represent more strains than observed in either assay in isolation, due to incomplete linkage of EBNA2 and LMP1 genes. This shows that EBNA2 and LMP1 HTAs are complementary to one another in strain detection.

### Stable persistence of virus in the three compartments

LMP1 and EBNA2 strains were recurrently detected in consecutive time-points in PBMC and saliva, and sometimes in plasma, in majority of the cases. EBNA2 HTA analysis of IM4 (Fig. 4A) showed that group 3e persisted in PBMC and saliva in all five time-points, while group 2 was observed in all longitudinal plasma samples. Similarly, C1 and C2 strains were identified by LMP1 HTA in PBMC, M+ and C2 in plasma, and C1 in saliva, in many of the time-points of IM4 (Fig. 4C).

Group 1, C1 and NC in PBMC, group 1 and C1 in saliva, and group 2 and M+ in plasma of IM4, persisted stably through multiple time-points. Group 3e, M+ and C2 in PBMC of IM6, group 2, M+ and C2 in plasma, and group 3e, C1, and C2 in saliva of the same case, again showed persistence through multiple time-points (Fig. 3).

**Fig 5:**
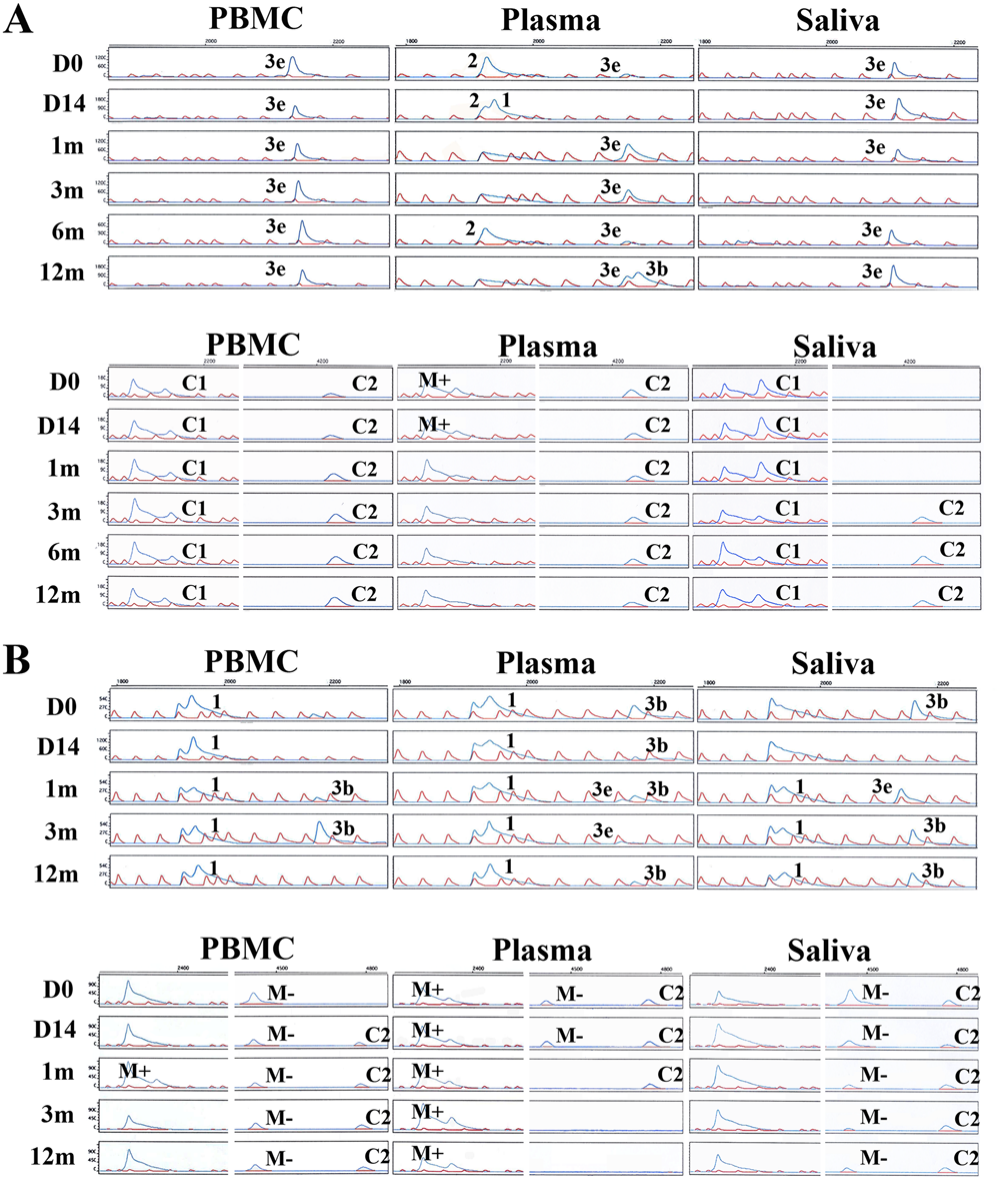
EBNA2 HTA and LMP1 HTA of IM10 and IM12. (A) EBNA2 HTA and LMP1 HTA of IM10 (B) EBNA2 HTA and LMP1 HTA of IM12. Red peaks represent the ROX ladder. The left, middle and right panel represents samples from PBMC, plasma and saliva, respectively. The strains detected were labeled beside the corresponding blue peaks. D0, D14, 1m, 3m, 6m and 12m represents time points of day 0, day 14, 1 month, 3 months, 6 months and 12 months, respectively. A summary of strains detected in this case is in Fig. 2 and 3.

In IM10 (Fig. 5A), all PBMC samples harbored C1 and C2 simultaneously, while in saliva, all samples were detected with China 1 only. Group 1 was found in all time-points of IM12 in PBMC and plasma (Fig. 5B), and in three out of five time-points in saliva. M- was detected in every time-points of PBMC and saliva of IM12, M+ in all plasma samples, and C2 in majority of samples in the three compartments.

Despite the general persistence of the dominant strain, minor strains were observed in most of the cases, either co-existing with or displacing the dominant strain at one or more time-points. For example, EBNA2 HTA analysis of IM7 showed that group 2 was predominant in plasma, yet at 1 month, group 1 was detected. LMP1 HTA analysis of IM1 found C1 and NC at day 0 or both PBMC and plasma. These two strains persisted in PBMC, yet in plasma it was replaced with M+, and this M+ became the dominant strain later. Relative abundance of a strain might vary across time, hence, it might become detectable at certain time-point and disappeared thereafter.

### Interchange of virus among different compartments

Interchange and transmission of virus among compartments was demonstrated by HTA in multiple cases. For example, group 3b appears first in plasma at 1 month of IM5 (Fig. 2), then in saliva at 6 months. This suggested a transmission of the virus from plasma to saliva. Since group 3e was detectable in PBMC and saliva samples collected at day 0, the group 3e strain observed at day 14 plasma sample might come from PBMC and saliva. LMP1 HTA showed that M+ was first detected in saliva at day 14, and appearing later at 6 months in PBMC. This suggested the M+ entered PBMC from saliva. In IM7 (Fig. 3), group 1 in plasma at 1 month might come either from PBMC or saliva, since group 1 is the dominant EBNA2 defined strain in these two compartments. By LMP1 HTA we also detected C2 at 12 months in saliva, not in previous time-points of saliva. PBMC and plasma can be the source of this C2. Occasional presence of group 1 in plasma of AS1, AS3 and AS7 might also due to transmission from PBMC and saliva. Assuming a virus is not eliminated from the body once infected a cell, emergence of a strain mid-way in our longitudinal study might indicate either a rise in relative abundance of a dormant strain, or transmission of this previously absent strain from other compartments.

### Concordance of viral strains in PBMC and saliva compartments

EBNA2 HTA performed on IM1, IM3, IM4, IM6, IM7, AS2, AS5 and AS7 displayed a close resemblance of strains detected between PBMC and saliva samples. Group 1 predominates in both PBMC and saliva of IM1, IM2, IM3, IM7, AS1 and AS7, while group 3e was observed in multiple time-points of PBMC and saliva samples of IM4, 5, 6, AS2 and AS5. Nevertheless, group 1 and 3e can also be detected in some of the cases in plasma, indicating that infection by these strains was not exclusive to the compartments of PBMC and saliva. The remaining subtypes, namely, group 2, 3a, and 3b, were detected as minor strains in PBMC and saliva. None of these five groups are exclusively found in any of the compartments.

EBNA2 HTA of IM4 (Fig. 4) illustrated that group 3e strain was present in almost all PBMC and saliva samples. Group 1 strain was detected only in the 12 month PBMC sample, but neither present in earlier time points nor in simultaneously collected saliva and plasma samples. Group 2 strain was only present in plasma samples of all time-points, not in PBMC or saliva.

IM1 and IM6 (Fig. 3) also show the difference between plasma and the other two compartments. Clear discordance of plasma from PBMC and saliva in IM1 is shown by EBNA2 HTA analysis. Group 1 is the only strain detected in PBMC and saliva, while only group 2 is observed in plasma. Similarly, LMP1 HTA of IM1 shows that C1 is mainly found in PBMC and saliva, while M+ is only found in plasma. Such discordance can also be observed in IM6 (Fig. 3). Group 3e is the dominant strain in PBMC and saliva, while group 2 is dominant in plasma.

### Greater diversity of viral strains in plasma

EBNA2 HTA of IM10 and IM12 revealed a great diversity of strains in the plasma compartment (Fig. 5). Group 3e was the only group found in PBMC and saliva of IM10 by EBNA2 HTA. In the contrary, four groups, group 1, 2, 3b and 3e, were detected in plasma (Fig. 5A). In plasma of IM12 (Fig. 5B), two groups, group 1 and 3b, were observed simultaneously in all time-point, except at 1 month, where there were three groups, group 1, 3b and 3e, co-existing. Only one to two strains were observed in PBMC and saliva of IM12. LMP1 HTA also showed three strains, M+, M- and C2, co-existing in first two time-points of plasma in IM12.

Four LMP1 strains, C1, C2, M+ and NC, were detected in plasma of IM8 (Fig. 2). Only C1 was detected in most of the time-points in PBMC and saliva of the same case. Three EBNA2 strains, group 1, 2 and 3b, were observed in plasma of AS7, yet only group 1 was seen in PBMC and saliva. Other examples which have three or more strains observed in plasma by either EBNA2 or LMP1 HTA analysis include IM2, IM7, IM9, IM11, IM12, AS1 and AS4 (Fig. 2).

### Validation of HTA results by Sanger sequencing

In order to validate the strains detected in EBNA2 and LMP1 HTA, sequencing analysis was performed on a subset of the EBNA2 and LMP1 nested PCR products. Cycle sequencing was performed in about 10% of the samples in both directions for verification. Sequences obtained from the selected samples were all concordant to the results of HTA.

LMP1 HTA performed on day 0 PBMC sample of IM12 showed that the heteroduplexes were identified at a position differ from the references. Sequencing analysis revealed four nucleotides different from the M- reference sequence. These non-synonymous nucleotide changes lead to amino acid changes of the translated protein at residues 335 (Gly to Ala), 352 (His to Arg) and 366 (Ala to Thr). Amino acid changes at residue 352 and 366 were reported in previous study as non-strain-determining changes in Mediterranean variant [34]. These mutations, therefore, represent the sequence variability of a strain within a population.

## Discussion

Typing assays and HTA were performed on 20 cases, which included 12 childhoodinfectious mononucleosis (IM) and 8 asymptomatic (AS) cases of primary EBV infection. Since there was no observable difference between the strain profiles of IM and AS subjects, these 20 cases can be considered as one cohort of EBV primary infection for the purpose of this study. The relative prevalence of types 1 and 2 EBV was in line with previous studies, which stated the type 1 EBV is prevalent in east and south Asia [3]. EBNA2 typing and LMP1 30bp deletion typing demonstrated that more than one strain can be present in a single sample. With the strength of identifying more than two genotypes at loci of interest, HTA provided additional useful information in this viral strain study. More importantly, since linkage between EBNA2 and LMP1 genes was not observed, the combination of EBNA2 and LMP1 HTA would provide greater discriminatory power on the detection of EBV strains than any single assay alone.

Presence of multiple strains was found in blood and saliva specimens collected simultaneously at the first time-point in the majority of the cases studied. These samples can be considered as a snapshot of strain profile at early stage of infection of that individual. Based on our current understanding of EBV biology, the virus transmitted mainly via saliva exchange. Saliva specimen, at any single time-point, was observed to rarely hold more than two viral strains. In the light of previous studies, showing preexisting host immunity to EBV might not necessarily protect against future EBV infections [35], it is possible that multiple strains were acquired from multiple carriers, with each carried one or two EBV strains, at the early stage of infection. In addition to EBV, multiple infections of human cytomegalovirus in healthy individuals [36] and Kaposi's sarcoma-associated herpesvirus in HIV-positive and negative individuals [37] were also reported, suggesting that multiple infection is a feature common in herpesvirus.

Consistent strains were observed in multiple time-points in our longitudinal study. The current model of EBV life cycle states that the growth program of the virus activates infected B cells to become proliferating blasts, they then differentiate into resting memory B cells through the process of the germinal-center reaction [38]. Some of these latently infected B cells will go into the peripheral circulation. The virus detected in PBMC, hence, represents a B-cell pool in circulation. On the other hand, the virus detected in saliva represents the epithelial pool, released from lytic replication of epithelial cells. The same virus strains were consistently detected in longitudinal samples of the same compartment implied that the two pools of virus persist stably throughout the one year of sample collection in our study. Despite the general consistency of detected strains through time, temporal changes in EBV strains were observed in sequentially collected specimens in the same individual. Due to the limitation of assay sensitivity, we could notprove conclusively the occurrence of sequential infection events. Emergence, disappearance and re-emergence of viral strains in our assays might reflect a change in relative abundance of co-infecting viral strains or an interchange of viruses among different compartments

Resemblance of strain profiles in PBMC and saliva reflected the intimate interaction between viruses released from the epithelial cells and circulating B cells, as part of the EBV life cycle. It is known that oral cavity and peripheral circulation are important anatomical sites of EBV infection and persistence. These two compartments are connected by orophayngeal lymphoid tissues such as the lingual, palatine, and pharyngeal tonsils. EBV-infected B-cells might re-enter the tonsils though the mechanism of lymphocyte homing, in which memory lymphocytes express characteristic sets of adhesion receptors in order to return to the target organs where they first encountered antigens [39,40]. EBV-infected cells can therefore release the viral particles through lytic replication, re-infect the cells in the lymphoepithelial tissues, and subsequently release viruses into the oral compartment[41,42]. Such an intimate and dynamic relationship allows EBV strains to equilibrate in these two compartments, hence, gives rise to close resemblance in the strain profiles.

Although infectious virions are also present in long-term virus carriers [43], attempts in typing EBV genome in plasma samples in healthy carriers were sometimes rendered futile due to low viral loads. Heightened viraemia in primary infection, though, provide us a chance to study the strains present in the plasma. Discordance of strains in plasma to those in PBMC and saliva was observed in a proportion of the cases. LMP1 HTA performed on Caucasian samples of primary infection patients also showed distinctive strains in plasma [26]. Since viruses harbored in multiple anatomical sites might be shed into the plasma, our findings might suggest that distinct viral strains can be harbored in hitherto undetermined tissue compartments. Indeed, EBV could be detected in different tissues at various anatomical locations of the human body [19].

In summary, this study indicated that multiple viral strains were acquired early at the start of infection. Dominant EBV strains were observed to persist stably in the same tissue compartment over time. Interchange of viruses between circulating B cell and epithelial cell pools is suggested by the concordance of EBV strains between PBMC and saliva, whilst discordant viral strains observed in plasma and PBMC/saliva is suggestive of the presence of distinct viral strains in hitherto undetermined compartments. Taken together, the results indicated that the distribution, persistence and interchange of viral strains among the tissue compartments are more complex than those proposed by the current model of EBV life cycle.

## Supporting Information

S1 Table EBV viral load in PBMC, plasma and saliva samples by quantitative PCR. (DOCX)
