# Supplementary material for: Distribution, Persistence and Interchange of Epstein-Barr Virus Strains among PBMC, Plasma and Saliva of Primary Infection Subjects
Source: PLoS One. 2015 Mar 25;10(3):e0120710. doi: 10.1371/journal.pone.0120710 (PMC4373854; doi:10.1371/journal.pone.0120710)
Supplement: S1 Table — (DOCX) [file pone.0120710.s001.docx]

**Supplementary data**

**Table S1. EBV viral load in PBMC, plasma and saliva samples by quantitative PCR**

| Case | Compartments | Day 0 | Day 7 | 1 month | 3 months | 6 months | 12 months |
| --- | --- | --- | --- | --- | --- | --- | --- |
| IM1 | plasma | 89435 | 4046 | 0 | 708 | 0 | 139 |
|  | PBMCs | 21523 | 1096 | 412 | 3015 | 2941 | 1908 |
|  | saliva |  |  |  |  | 24240667 | 2693333 |
| IM4 | plasma | 736 | 237 | 129 | 60 |  | 0 |
|  | PBMCs | 1676 | 786 | 659 | 2104 |  | 37 |
|  | saliva | 7294000 | 97701 | 7549267 | 2911200 | 198385 | 103944 |
| IM5 | plasma | 583827 | 3185 | 1367 | 182 | 0 |  |
|  | PBMCs | 157903 | 1135 | 7212 | 4277 | 122 |  |
|  | saliva | 115161 | 3338 | 3557 | 29052 | 11750 | 0 |
| IM6 | plasma | 902780 | 455 | 617 | 114 | 113 | 0 |
|  | PBMCs | 162217 | 1631 | 5861 | 3384 | 1243 | 377 |
|  | saliva | 5165533 | 61704667 | 547780 | 914020 | 499933 | 1351493 |
| IM7 | plasma | 252567 | 1432 | 808 | 292 |  | 0 |
|  | PBMCs | 146338 | 564 | 2244 | 3342 |  | 171 |
|  | saliva | 106302000 | 11047500 | 19313667 | 7124333 | 1432600 | 15213 |
| IM8 | plasma | 46063 | 545 | 343 | 386 |  | 0 |
|  | PBMCs | 4215 | 100 | 793 | 5758 |  | 1336 |
|  | saliva |  | 1402 | 2302 | 518027 | 561873 | 388927 |
| IM9 | plasma | 1004620 | 1379 |  | 891 |  | 542 |
|  | PBMCs | 347419 | 4068 |  | 22879 |  | 623 |
|  | saliva | 1937553 | 26776667 | 18229000 | 929753 | 464853 | 436553 |
| IM10 | plasma | 5605 | 166 | 0 | 0 | 404 | 394 |
|  | PBMCs | 1057 | 490 | 5443 | 404 | 130 | 96 |
|  | saliva | 110964667 | 119262000 | 199188667 | 6213 | 395107 | 2954467 |
| IM12 | plasma | 122152 | 250 | 0 |  | 0 |  |
|  | PBMCs | 66853 | 1192 | 14400 |  | 2325 |  |
|  | saliva |  | 134107 | 2161040 | 649847 | 1721 | 96026 |
| AS1 | plasma | 1080 |  |  | 1445 | 0 | 0 |
|  | PBMCs | 1212 |  |  | 5883 | 597 | 212 |
|  | saliva | 4867 |  |  | 7660 | 5091 | 1850140 |
| AS3 | plasma | 0 | 932 | 520 | 0 | 0 | 0 |
|  | PBMCs | 1198 | 2649 | 452 | 1167 | 697 | 283 |
|  | saliva |  |  |  |  | 486 | 82723 |
| AS4 | plasma | 1684 | 471 |  | 0 |  | 0 |
|  | PBMCs |  | 438 |  | 46 |  | 132 |
|  | saliva |  |  |  |  | 253 | 83592 |
| AS5 | plasma | 0 | 412 | 212 | 800 | 659 | 189 |
|  | PBMCs | 66 | 106 | 452 | 1059 | 608 | 534 |
|  | saliva |  |  | 33310667 | 2091220 | 3673533 | 13118 |
| AS6 | plasma | 330 | 0 | 73 | 0 |  |  |
|  | PBMCs | 0 | 471 | 601 | 99 |  |  |
|  | saliva |  | 341 | 17473 | 375 | 0 |  |
| AS7 | plasma | 1587 | 118 | 8361 | 1435 | 0 | 66 |
|  | PBMCs | 2657 | 1241 | 1449 | 4657 | 1725 | 1160 |
|  | saliva |  | 91489333 | 23375333 | 29583333 | 2414800 | 462404 |

*PBMC viral load is in copies per million cells; plasma and saliva viral load is in copies per ml plasma or saliva
